# Complicating the Story of Popular Science: John Maynard Smith’s “Little Penguin” on *The Theory of Evolution*

**DOI:** 10.1007/s10739-019-9566-y

**Published:** 2019-05-15

**Authors:** Helen Piel

**Affiliations:** 1 School of Philosophy, Religion and History of Science, University of Leeds, Woodhouse Lane, Leeds, LS2 9JT UK; 2 Contemporary Archives and Manuscripts, Politics and Public Life, The British Library, 96 Euston Road, London, NW1 2DB UK

**Keywords:** John Maynard Smith, Popular science, Evolutionary theory, Science communication, Neo-Darwinism

## Abstract

Popular science writing has received increasing interest, especially in its relation to professional science. I extend the current scholarly focus from the nineteenth to the twentieth century by providing a microhistory of the early popular writings of evolutionary biologist John Maynard Smith (1920–2004). Linking them to the state of evolutionary biology as a professional science as well as Maynard Smith’s own professional standing, I examine the interplay between author, text and audiences. In particular, I focus on Maynard Smith’s book *The Theory of Evolution* (Penguin 1958) and show how he used it to both promote neo-Darwinism and advocate the utility of mathematics in biology. Following in the footsteps of Charles Darwin and David Lack, Maynard Smith was a science communicator blurring the lines between genres (popular, professional, textbook) and audiences (expert and non-expert) while contributing to ongoing discussions within and on the profession of evolutionary biology around the Darwin-Wallace centenary.

## Introduction

The constantly changing relationship between professional and popular science increasingly receives a lot of scholarly attention. The diffusionist or deficit model, a prominent view of popular science or science popularization, supplies an undynamic view in which scientific elites produce knowledge which trickles down to a passive lay public. This model assumes two separate spheres in which scientific knowledge travels only one way. In a now-classic article, Cooter and Pumfrey (1994) pointed out the problems with historians of science’s mostly uncritical acceptance of this model and similarly the implied dichotomy of elite versus popular cultures (pp. 248–252). They called for a re-thinking of the history of popular science, bringing in “the essentially dialectical basis of the construction of popular culture” (p. 252). Similarly, Stephen Hilgartner has problematized the diffusionist model; he prefers to think of science popularization as happening on a continuum and in degrees (Hilgartner 1990; see also Whitley 1985; Myers 2003; and Bucchi 2008).

Indeed, we locate arguments doubting the view that science popularization is either unimportant to or isolated from professional science as far back as 1935. Ludwik Fleck’s monograph on the emergence and genesis of scientific facts, translated into English in 1979, expressed a view that includes feedback loops between popular and professional science and literature. He stressed the importance of popular literature for the formation of the worldviews of scientists and communication not only between scientists and non-scientists but also between scientists of different (degrees of) specializations. Popular science thus forms the basis of every person’s knowledge. However, Fleck problematically also characterized popular science as defined by loss of detail and controversy, leading to artificial simplification, and simple acceptance or rejection of certain points of view, with the aim to create a worldview.

As concepts, both popular science and science popularization have been criticized. Associating popular science with the diffusionist model contributes to this particular area of critique. Therefore, James Secord suggests to re-conceptualize popular science as part of a broader “knowledge in transit” category: “to think, at every point in our work, about science as a form of communicative action—to recognize that questions of ‘what’ is being said can be answered only through a simultaneous understanding of ‘how,’ ‘where,’ ‘when,’ and ‘for whom’” (2004, p. 663f). Moreover, Jon Topham keenly “advocate[s] a closer attention to actors’ own categories of the ‘popular’” (2009a, p. 5). At least part of the appeal of Secord’s focus on communication and Topham’s attention to actors’ categories is that:

the fact that “popular science” has been used by actors and historians alike to refer variously to science *for* the people, the science *of* the people, and science *by* the people ceases to be a problem. All of these are considered legitimate objects of historical inquiry, contributing to a common project of understanding how knowledge comes to be constituted and reconstituted within culture. It is this that makes the history of “popular science” a central aspect of the history of modern science. (Topham 2009b, p. 317)

In a focus section in *Isis*, Andreas Daum points to several other problematics, highlighting imbalances in scholarly focus. These imbalances were emphasis on (natural) science, on English-speaking literature and Britain, and on the nineteenth century (2009, pp. 322–323). Ralph O’Connor similarly highlighted that “the postwar period [is] currently little studied by historians” (2009, p. 335). As Topham (2009a) and others have pointed out, popular science emerged in the nineteenth century, which accounts for the scholarly focus on this period. The nineteenth century indeed offers rich case studies about early stages in this development. At the same time, there is a long-standing view that with increasing professionalization in the twentieth century, popular science lost its standing. But, as Peter Bowler has indicated, scientists continued popularizing throughout the early twentieth century. Among others, Bowler mentions Penguin’s Pelican series as an effort to share scientific knowledge with an engaged readership (2009, 2006).

This paper will address these chronological imbalances and offer a first step towards a history of twentieth-century popularizers through a microhistory of John Maynard Smith, FRS (1920–2004) and his early works in science communication, in particular his *Theory of Evolution* (Penguin 1958). Such microhistories are necessary, as Bernadette Bensaude-Vincent has reminded us, to “test the hypothesis” of these more recent interactive models of popular science (2009, p. 367). Indeed, this paper joins the emerging view that the relation between popular science, professional science, and science professionalization is more complicated than sometimes appreciated (e.g., Smocovitis 2014, p. 111): in Maynard Smith’s early popularization work, we find a genre-bending, multi-layered, and audience-bridging work of science communication. This paper also reflects on the argument that when scientists did popularize, they often did so after their own professional status was secure and continued to keep their proper science and popular science separate (see Ruse 1996, 1999 on Theodosius Dobzhansky and Julian Huxley; Lightman 2007 on T. H. Huxley and Robert Ball).

## “Birds as Aeroplanes” (1953): Maynard Smith Enters Popular Science

Maynard Smith was one of Britain’s most eminent evolutionary biologists and winner of, *inter alia*, the Crafoord Prize (biology’s equivalent to the Nobel Prize). The prize was jointly awarded to Maynard Smith, Ernst Mayr, and George C. Williams in 1999 for “their pioneering contributions to broadening, deepening and refining our understanding of biological evolution and related phenomena such as the formation of species and their adaptation to changes in their environment.”^1^ His career spanned half a century. After leaving his first career as an aircraft engineer in the 1940s, Maynard Smith became a biologist, continuing until his death in 2004. As a theoretical biologist with mathematical expertise, he worked on the evolution of sex and of senescence, but he is best-known for his development and introduction of evolutionary game theory and of the evolutionarily stable strategy (ESS).The British public voted his work the 61st-most important British innovation of the 20th century.^2^ The majority of his career was spent at the University of Sussex’s School of Biological Sciences, of which he had been appointed founding dean in 1965 and from which he retired in 1985. Retirement did not mean an end to his career, however. Maynard Smith was actively researching and publishing until right before his death; his last book, *Animal Signals* (co-authored with David Harper) was published in 2003.

As indicated, Maynard Smith initially studied engineering; he had a degree from Cambridge and worked as an aircraft stressman during World War II before he read zoology at University College London under J.B.S. Haldane (Kohn 2004). During his early career, he was interested in animal locomotion and published his first scientific paper on “The importance of the nervous system in the evolution of animal flight” (1952). This paper and research conducted earlier with a fellow undergraduate student, M. J. Davis, formed the basis for Maynard Smith’s first popular publication, “Birds as aeroplanes” (1953). It was published in Penguin’s *New Biology* series, then one of only few popular biology publications (Bellairs 2000, p. 26). The series’ editors, Michael Abercrombie and M. L. Johnson (zoologists at the University of Birmingham), emphasized in the first volume that the series was one of “serious science” and “not light reading” (Johnson and Abercrombie 1946, p. 7). Yet (over)simplification was avoided; Abercrombie and Johnson hoped readers would be stimulated and take the initiative to look up background information. They were aware that their current target audience of those “already possessing some scientific knowledge, whether through self-education or through school or university instruction” was “small now,” but that it was “certain to grow enormously before long” (p. 7). Not 10 years later, the Minister of Education appointed a committee to review adult education opportunities in Britain. Chaired by Dr. Eric Ashby, the committee concluded “in relation to the community at large, adult education students represent a social and intellectual asset the loss of which would be deplorable” (Anonymous 1954, p. 865). Adult education was acknowledged as an important value that needed support. The report focused mainly on universities’ activities and the Workers’ Educational Association, but also adult education, which took place via popular science as presented in books, magazines, and museums, later joined by radio and television in audio and visual formats (see, for example, Boon 2008; Jones 2010). Natural history museums had been growing in popularity since the late 1920s (Rader and Cain 2014, p. 113). Maynard Smith himself visited the London Natural History Museum as a child before moving to the countryside (Maynard Smith 1985, p. 347).

Maynard Smith’s publication in a popular science journal and his later popular science book need to be situated within the context of his continued interest in adult education and the popularity of biological topics. In a country of birdwatchers, his first popular article, “Birds as aeroplanes” (1953), enjoyed wide readership. David Lack of Oxford University arranged its publication after hearing Maynard Smith talk at an ornithological meeting. This is important for two reasons. First, for Maynard Smith this was “a welcome success”; he was encountering difficulties publishing work in which he was applying mathematics to biology (Kohn 2004, p. 214). “Birds as aeroplanes” was accompanied by a short biography to establish his status as an expert, pointing to his careers as aircraft engineer and as lecturer at UCL (Anonymous 1953, p. 127). Penguin’s Pelican imprint had started adding author biographies around the 1940s, a necessity since at the time, many of their original works were “mostly written by lesser-known experts” (Bowler 2009, p. 266)—as Maynard Smith was at the time.

Second, Lack himself had experience of writing popular science books which do not fit the diffusionist model. His *Life of the Robin* was first published in 1943 by H. F. & G. Witherby and—important for the connections between the scientists and publishers in this story—re-published in 1953 as a Pelican paperback. Lack knew whom to approach at the publishers and what they were looking for. *The Life of the Robin* was both scientifically rigorous and readily accessible by combining “scientific description and analysis with literary and historical references to the subjects discussed” (Anderson 2013, p. 25); his popular science was as much science as it was literature. Lack’s work fits Ralph O’Connor’s (2007) “science *as* literature,” rather than “science *and* literature,” approach to popular science. With his later *Theory of Evolution*, Maynard Smith explicitly placed himself in the tradition of biologists like Lack who were writing books accessible to and read by both experts and non-experts in their fields. Adding “Further Reading” suggestions to his book, Maynard Smith lists Lack alongside Charles Darwin, Theodosius Dobzhansky, J. B. S. Haldane, Julian Huxley, Ernst Mayr, George G. Simpson, and others (Maynard Smith 1953, p. 306).

“Further Reading” suggestions were not part of the article Lack helped Maynard Smith publish in 1953, but in its focus on birds their common interest is obvious. “Birds as aeroplanes” notably combined Maynard Smith’s two skillsets biology and engineering by discussing wings, control of direction and speed, types of unpowered flight (soaring and sailing), and the source of power for flight (Maynard Smith 1953, p. 64). Of particular interest and importance are the sections built on work not in the 1952 publication, since they indicate two aspects of Maynard Smith’s writing both in his popular and professional works: familiar references and mathematics. When discussing flight without the flapping of wings, he at first referred to vultures and albatrosses, pointing out the differences in their wings and structures. Vultures use columns of warm air to gain lift; they soar. Albatrosses, in contrast, rely on different horizontal wind velocities over sea; they sail.

A similar, though less extreme, contrast can be seen in this country between, say, a buzzard and a herring gull; it was in fact the sight of these two birds in the air at the same time, one inland and the other out to sea, which first drew my attention to this difference in structure, and led me to speculate on the reason for it. (Maynard Smith 1953, p. 72)

Replacing the exotic with the familiar helps Maynard Smith evoke a much clearer image in the mind of his audience. Maynard Smith would stay true to this way of presentation in his book: “I have not assumed any specialized knowledge in the reader, and when possible have drawn my examples from familiar animals and plants” (Maynard Smith 1958, p. 12).

The second feature mentioned is the use of mathematics. A trained engineer, he was not afraid of approaching biological problems mathematically. Other biologists were less at ease with equations, evidenced in the rejection of several of Maynard Smith’s early papers. His first scientific publication had not included any mathematics either, but “Birds as aeroplanes” confronted its reader with three equations and, in a footnote, invited “[m]athematically minded readers” to “prove this statement”—and assured “[t]hose who do not like mathematical arguments … that the statement fits the observed facts” (Maynard Smith 1953, p. 72).

“Birds as aeroplanes” was a stepping stone for Maynard Smith and highlights some important elements. It allowed him to publish research he could not publish elsewhere—his work with Davis had been rejected earlier (Maynard Smith 1997)—and there seems to have been less hesitation or less strict rules about the inclusion of mathematics. But *New Biology* not only offered a platform for Maynard Smith, an early-career researcher, to get his ideas into the public domain. It also established him as an expert suited to communicating scientific ideas, placing him alongside reputable scientists publishing in the same journal and further setting up his credentials with the accompanying biographical entry. This expertise by association is repeated, more actively, through the mentioned “Further Readings”; Maynard Smith chose which works to include, linking their authors’ expertise to his.

The view that popularizing scientists were damaging their career therefore does not hold in Maynard Smith’s case. Bowler has already painted a more nuanced picture: the scientists who seem to have been derided for their popularizing efforts—which includes Maynard Smith’s mentor and fellow-publisher in *New Biology,* J. B. S. Haldane—were writing for the daily press. The scientific community was generally not objecting to scientists writing educational material of a more “serious” nature. *New Biology* cannot be compared to the *Daily Worker* (another of Haldane’s outlets), and “[p]rovided one kept such activity limited to a level where it still left plenty of time for research, [popularization] was welcomed rather than criticized by the majority of scientists” (Bowler 2006, p. 163). As Bernard Lightman has noted, despite continuities from the nineteenth century in terms of traditions which “continue to shape the way science is popularized and the way that current audiences consume it” (2007, p. 498), the increased professionalization of science did make a difference to popularization in terms of who wrote what and when, and how they were viewed by the scientific community. He echoes Bowler’s analysis that “[e]minent scientists could retain the respect of their peers when they took on nonspecialist writing as long as they had made a substantial contribution to research at the same time” (p. 419). The important anomaly in the case of Maynard Smith is that in the 1950s he was not yet an eminent scientist. He had started establishing himself as a fruit fly geneticist, but the work he is best-remembered for, evolutionary game theory, was not done until the 1970s.

## *The Theory of Evolution* (1958) as Professional Popular Science

Shortly after his first venture into science communication to a non-specialist audience, Maynard Smith began a second project. On 16 February 1956, he signed a contract with Penguin’s Pelican imprint to deliver “a literary work at present entitled: THEORIES OF EVOLUTION”).^3^ The book was to be the first volume in the new Pelican Biology Series. The editors and Maynard Smith aimed high, wanting to cover everything relevant to the theory of evolution, which required knowledge in many different fields of biology (Maynard Smith 1958, p. 11). This lofty goal fit into Pelicans’ aim of combining “intellectual authority with clear and accessible prose” at an accessibly low price (6d.): “an informal university for generations of Britons.”^4^

Unsurprisingly, Maynard Smith took up this challenge. His own early science education was primarily based on reading popular science in Eton’s school library: Haldane, Huxley, and Eddington, as well as Darwin, Einstein, and Marx (Maynard Smith 1985, p. 347). These were not necessarily popular science books in terms of books simplifying science for the non-expert; in fact, when he came across these works, Maynard Smith mostly found them rather dissatisfying as he “always had the feeling that difficulties were being slurred over” (1958, p. 12). He wrote *The Theory of Evolution* in a style reminiscent of nineteenth-century popularizers, aiming at multiple audiences as well as twentieth-century biologists whose books could be and were read by both experts and non-experts, whether envisaged as having popularizing potential or not.

As noted above, Maynard Smith added twelve authors to his “Further Reading” suggestions, books that had inspired him and others in their studies of evolution: Darwin, Huxley, Haldane, and Lack among them. Lack was partly inspired by Haldane’s *The Causes of Evolution* (Lack 1973, p. 425), the work on Maynard Smith’s list. Maynard Smith’s suggested text by Lack was the work responsible for indelibly linking Darwin and his evolutionary theory to birds in the public eye—*Darwin’s Finches* (1947)*. “*Just over a hundred years ago, in 1835,” Lack wrote:

Charles Darwin collected some dull-looking finches in the Galapagos Islands. They proved to be a new group of birds and, together with the giant tortoises and other Galapagos animals, they started a train of thought which culminated in the *Origin of Species*, and shook the world. (Lack 1961, p. v)

This text made Lack world-famous and was a book “by a writer today” to be “class[ed] with the works of Darwin” (Hardy 1973, p. 435). This and the other books all on Maynard Smith’s list “made important and original contributions to our understanding of the causes of evolution.” And while they were for the most part, at least originally, aimed at professionals, Maynard Smith noted they could “be read with profit by a layman” (Maynard Smith 1958, p. 306). Other books on the list, Darwin’s included, were consciously aimed at both the public and his scientific colleagues (Lightman 2010, pp. 7–9). Darwin was not the only nineteenth-century scientist using a book aimed at multiple audiences to promote a scientific, theoretical point; William Buckland’s Bridgewater Treatise on geology addressed “a range of intended audiences” and inspired “a range of often unintended usages.” Importantly, “[w]riting the book for a wide audience gave Buckland licence to provide an overview of the subject which was of considerable importance for practitioners in his own scientific field” (Topham 2009a, pp. 17, 18).

Such an overview was also the aim of *The Theory of Evolution* and the Pelican Biology series. In a sense, the book was a summary of the ideas presented in the more professional literature of the “Further Readings,” written in the hope that it “will be of value to the non-specialist in summarizing a set of ideas which, taken together, form perhaps the most important contribution yet made by biologists to our understanding of what the world is like and how it came to be like that” (Maynard Smith 1958, p. 11). That it had potentially the same use for experts—similar to Buckland’s work—became clear later in the writing process and was acknowledged by both Maynard Smith and the publisher. Initially, the book was simply meant to start and lay the foundation for the Biology series. Abercrombie and Johnson, in 1953 (the same year they published “Birds as aeroplanes”) had prepared a scheme for the Pelican Biology series. Comparable to the Pelican History of England, the “idea is to have … a set of volumes covering substantially the whole range of biological sciences … which people will buy with the feeling that they are getting a fairly systematic survey of the field.”^5^ They suggested ten topics: nature of life, history of life, modern evolution theory, reproduction and life history, population and communities, parasitism, physiology of animals and plants, nervous system and behavior, biology and human affairs, and lastly the discovery of modern biology. Concerning Maynard Smith’s topic “Modern Evolution Theory,” Abercrombie and Johnson highlighted the recent advances that were “still substantially unknown to ordinary readers.”^6^ As with their *New Biology* series, they wanted to introduce non-specialists to a variety of important biological problems and thoughts. Highlighting the novelty of the scientific knowledge to be presented made sense both considering their educative outlook and as a pitching strategy to publishers and prospective authors.

Maynard Smith submitted his outline in 1955. Abercrombie was delighted, noting: “he seems to have thought his subject out afresh” and there was “little doubt that we shall get an excellent book from him”:

I know that it looks a bit formidable and perhaps a bit long; Smith, however, realizes well enough the sort of audience he is writing for and I will keep him to it. He has merely written the headings out in rather technical language.^7^

Almost all the headings did make it into the published book, however.^8^ Maynard Smith used scientific terminology in both titles and text; he saw no need to either dumb down or sensationalize his language for the benefit of the reader. On the contrary, his use of a matter-of-fact style only emphasized the blurring of traditional boundaries found in the dominant view of science popularization. Maynard Smith *was* aware of his audience, but he was also aware that this audience was multifaceted and engaged. Ultimately, his book even bridged the genres of popular science and textbooks, being advertised as the former but then also used as the latter.

In 1956, Maynard Smith signed his contract. A small but important change to the book’s title between then and its publication in 1958 reflects Maynard Smith’s view of the state and status of evolutionary biology at the time. “Although I agreed to write a book on ‘THEORIES OF EVOLUTION’ I did not in fact discuss any theory of evolution other than Darwin’s, so I would prefer the title ‘The Theory of Evolution.’”^9^ According to Maynard Smith, there is only one justified view on evolution. The original title was inclusive, pluralistic, and referring to an undefined number of “theories,” but as Maynard Smith explained in the introduction: “The main unifying idea in biology is Darwin’s theory of evolution through natural selection.” The new title thus better supports the overall perspective that “recent advances in these various fields (of biology) … can only be fitted together to tell a coherent story if the theory of natural selection is accepted” (Maynard Smith 1958, pp. 11, 83).

Another less explicit but connected reason for the change lies in the general confusion between what scientists and non-scientists associate with the word “theory.” When a biologist “calls evolution a theory, he means it is a central disciplinary concept enabling further thinking about life. When the *Times* calls it a theory, the connotation is that it is another airy idea dreamed up by scientist” (Myers 1990, p. 190). Using plural “theories” in the title of a book that was meant to depict a coherent story of a proper science would have only made matters worse or at the very least offered fuel to any sceptics of neo-Darwinism which was also known as “the modern synthesis.” Neo-Darwinism describes the biological perspective represented by Maynard Smith in *The Theory of Evolution*:

By Darwinism is meant the idea that evolution is the result of natural selection. Neo-Darwinism adds to this idea a theory of heredity. In its most general form, the theory of heredity is Weismannism, that is, it is the theory that changes in the hereditary material are in some sense independent of changes in the body…. In particular, the theory of heredity is Mendelian, that is, it assumes that heredity is atomic, and obeys either Mendel’s laws or some modification of them explicable in terms of the behaviour of chromosomes. (Maynard Smith 1972, p. 82)

By using *The Theory of Evolution*, Maynard Smith succeeded in “providing non-specialists with a systematic survey of the present state of biological theory” (E.H. 1958, p. 572). It was more than a survey though; he presented a strong neo-Darwinian interpretation of biological theory (despite including exceptions to the rule). As David Sepkoski (2014) has noted regarding the paleobiology debates of the 1980s, the reasons why scientists write popular science are manifold, and one can be to package as popular writing concepts and theories really meant to convince professional peers.

To provide this survey and convince readers of neo-Darwinism, Maynard Smith begins with the basics: adaptation, the theory of natural selection, heredity, and variation. After introducing the reader to these concepts and their role in evolutionary biology, he covers broader issues such as species, patterns of evolution, and the evolution of major groups. Throughout the work, he discusses pros and cons of specific ideas and theories within evolutionary biology; chapter 16, for instance, examines Richard Goldschmidt’s arguments against a neo-Darwinian view of speciation (the idea of “hopeful monsters” with which Maynard Smith ultimately disagrees) before moving on to C. H. Waddington’s notion of canalization (which he says needs more research). Maynard Smith’s neo-Darwinian conviction and training are evident: he was a staunch supporter of adaptation and natural selection, and adaptation, for him, was *the* biological problem that needed solving (Kohn 2004, p. 225). Here is a strong example of popular and professional science interacting; Maynard Smith invites the public to engage with discussions which were still ongoing among evolutionary biologists and which, importantly, are not resolved even though he suggests his own interpretations of the validity of the ideas he presents.

Taken together with the “Further Reading” list, the invitation Maynard Smith extends allows a certain amount of agency for the reading audience (and again proves that we need a more nuanced notion of popular science in that, by communicating uncertainty, it undermines the notion of top-down dissemination of certain knowledge). They are being taken seriously rather than talked down to. Indeed, from the beginning, Maynard Smith was not afraid of presenting his readers with difficult concepts, ideas, or mathematics. “I have not omitted any subjects merely because they are difficult,” at the risk “that some parts of this book will prove rather hard going” (Maynard Smith 1958, p. 12). These parts include much of Maynard Smith’s own research in genetics. By the end of the 1950s, he had published a dozen papers on *Drosophila* research. Fruit fly genetics had been famous since T. H. Morgan’s “Fly Room” at Columbia University, and *Drosophila* had proven to be very good for genetic research (Sturtevant 2001). They have the advantage of being small enough to be stored in large quantities yet still large enough so that physical changes can be observed easily enough; they breed quickly and have large amounts of offspring; and the large chromosomes in their salivary glands are observable through the microscope (Maynard Smith 1957, p. 85; Jennings 2011; Hine 2015, p. 183).

The experiments done by Maynard Smith, Morgan, and others are covered in chapter 5 on “Artificial Selection: Some Experiments with Drosophila.” Maynard Smith gives the reader case studies of laboratory work—artificial selection—to demonstrate *if* and *how* natural selection acts on populations in the wild in the next chapter (Maynard Smith 1958, p. 83). While there is no one-to-one translation of controlled laboratory experiments to nature, we can still learn from such experiments. Firstly, *Drosophila* research has shed much light on general principles of heredity. Studies of bristle numbers in subsequent generations of fruit fly, artificially selected for either more or fewer bristles, have answered questions about the development of frequencies of individuals with different characters over time, about how closely relatives will resemble each other, and about what happens if artificial selection for one character is done to the extreme. Three conclusions are drawn from the bristle studies:

- selection would at first lead to a rapid change in the population mean in either direction;
- progress under selection would slow down, and finally stop, because there would no longer be any genetic variability for which we could select; and
- at this final stage, the population would be much less variable than the initial population. (Maynard Smith 1958, p. 89).

In effect, extreme selection leads to diminished genetic variability for selection to work with. This would also hold true for wild populations—unless there were some processes to bring about new variations to break the cycle. Maynard Smith builds a bridge from the genetics-heavy chapter on selection to the chapter on “The Origins of New Variation.” While there is no explicit talk of genetics (as is the case in the previous chapter), the links between natural selection and the need for new variations are unmissable if both chapters are read, and Maynard Smith helped readers with a six-step summary of the genetics argument. The importance of the issues discussed becomes clear in combination with the explanation of how variations arise (and Maynard Smith does confine himself to genetic differences) and why they are important: “No one can claim to understand how evolution works without some basic understanding of classical population genetics” (Charlesworth 2015, p. 667).

Maynard Smith also brings home his main argument: that evolutionary changes are adaptive, not accidental (Maynard Smith 1958, p. 120; see also Drake 2007, p. 6). Genetic differences arise by accident—say, by mutation—and are often harmful. Natural selection, however, acts on these changes and thus brings about “continuous, adaptive, and seemingly purposive evolutionary changes” (Maynard Smith 1958, p. 120).

We have seen a first emphasis on natural selection in Maynard Smith’s discussion of genetics. Natural selection is the unifying principle for both the book and the theory of evolution, and it is naturally evident from the very beginning of *The Theory of Evolution*. The reader is asked to follow a mathematical model of a mouse population as early as chapter two, “The Theory of Natural Selection.” Maynard Smith introduces us to 100 dark and 100 light-colored mice. Over the next couple of pages, owls, disease, and other natural forces diminish subsequent generations of the original population; survivors then breed with each other. (We therefore have a simplified model population—breeding occurs once per cycle only, so that the numbers are easier to follow.) Maynard Smith makes the following point: the owls eat more light-colored mice which show up on the ground more easily, while the other forces kill an equal number of mice in both populations irrespective of coat color. Thus, the model explains the effects of natural selection on a population (Maynard Smith 1958, p. 34). The example illustrates Haldane’s notion of “intensity of selection,” which gives “a measure of how many lives are lost because not all individuals are as well adapted as are the fittest members of the population” (ibid., p. 36). At the same time, and not dissimilar to the laboratory experiments with *Drosophila*, the owl and mice example functions as a way to introduce larger concepts of natural selection. This allows for examples of natural selection working in the wild (e.g., the peppered moth) to follow in later chapters. In both cases, with the fruit flies and the mice, Maynard Smith first introduces his audience to a version in which parameters and variables can be controlled. Notably, this does not constitute a simplification of “real science.” Instead, these controls mirror a regular practice among scientists. After understanding concepts or processes based on laboratory experiments or mathematical models, it is then possible to apply these practically or theoretically to populations in the wild. Similarly, Maynard Smith moves from these “simplified” examples to research carried out in the field. He reintroduces the complications of uncontrolled variables and general complexity to demonstrate how natural selection works in the wild.

With the population model, we also have a first appearance of mathematics. There is a still-present notion that equations in popular science books are a serious problem for sales.^10^ Maynard Smith had trouble publishing because of his inclusion of equations, but this was a larger problem within professional journals more so than in his popular writings. Thus, mathematics reappears “at the risk of irritating readers who dislike even the simplest algebra” in Maynard Smith’s discussion of the Hardy–Weinberg ratio (Maynard Smith 1958, p. 125). The formula is a simple way of mathematically explaining why gene proportions, or frequencies of genotypes, stay stable—in equilibrium—in a population. It is a “general rule that holds when there are different, indeed varying, proportions of alleles floating around in a population” (Depew and Weber 1995, p. 232). Maynard Smith uses the equation to illuminate aspects of natural selection in wild populations, and in particular, on the question of industrial melanism (the peppered moths). A table illustrates the mathematics, and Maynard Smith’s explanation is an example of his clear use of language:

Suppose that there are two alleles, *A* and *a*, at a particular locus, and that their frequencies in a population are *p* and *q* respectively, where *p *+ *q *= 1. If, for example, *A* were nine times as common in the population as *a*, then *p* would be 0.9 and *q* 0.1. The probability that an individual receives the allele *A* from his father is then *p*. If mating is random, there is a similar chance *p* that he also receives an allele *A* from his mother. Hence the chance of an individual receiving *A* from both parents is *p* x *p *= *p*^2^, which is therefore the proportion of *A/A* individuals in the population. By an exactly similar argument, the proportion of *a/a* individuals is *q*^2^, and of *A/a* (or *a/A*) individuals is *2pq.* (Maynard Smith 1958, p. 125)

To the mathematician G. H. Hardy, this generalized formulation of how the Mendelian scheme would affect populations of interbreeding individuals, had “seemed so self-evident that he commented: ‘… I should have expected the very simple point which I wish to make to have been familiar to biologists’” (Sturtevant 2001, p. 107). Maynard Smith would have shared Hardy’s feelings about the mathematical incompetence of some biologists. For him, mathematics plays a vital role in biology: “natural history without mathematics is muddled” (Maynard Smith 1982; see also Maynard Smith 2002, p. 193).

## Placing the Penguin in Context: Professional Biology 100 Years After Darwin and in the Wake of the Modern Synthesis

Being published around two major centenaries for the theory of evolution—the publications of Darwin and Wallace’s paper proposing natural selection as an evolutionary mechanism in 1858 and of Darwin’s *The Origin of Species* the following year—Maynard Smith’s celebration of evolution by natural selection is no surprise: “It is appropriate that the first volume of Pelican Biology, coinciding with the Darwin-Wallace centenary, should be about the theory of how evolution occurs, one of the aspects of scientific knowledge that biologists can take most pride in” (Abercrombie 1958, p. 9). As a student of Haldane’s, Maynard Smith’s neo-Darwinian take is equally unsurprising, and indeed the only way to go, according to Julian Huxley’s review of the book (Huxley 1958). Yet, if one widens the view to the centennial literature as a whole—and there was a lot of it—a different view emerges.

In his review of 1958/59 publications, historian Donald Fleming registered surprise “that many of the writings display a distinct animus against Darwin or natural selection or both” (Fleming 1959, p. 439). There was a general misunderstanding of what Darwin actually achieved: many authors failed to understand that it was not the idea *of* evolution, but the idea of natural selection as a mechanism *for* evolution which was so ground-breaking. In a sense, the scientists did better justice to Darwin in placing him and his research historically than many historians, and Fleming recommended not only Maynard Smith’s book but also works by R. A. Fisher, H. B. D. Kettlewell and Julian Huxley. Bert Loewenberg, another reviewer perplexed by the ungenerous views of Darwin, agreed with Fleming, concluding the biologists “have not only succeeded in summarizing the evidence with a clarity rare among the technically expert, but they have analyzed the data in the perspective of significance” (1959, p. 529). Maynard Smith is one of the biologist writers deserving “centennial laurels” for his discussion of both seminal and contemporary state-of-the art research in evolutionary biology. This was a feat unrivalled at least until the early 1990s, when *The Theory of Evolution’*s re-issue stated that there still “is no other account of evolutionary biology available which is at the same time written for a non-professional readership, and which covers the whole field” (Maynard Smith 1993, p. 1).

Maynard Smith’s survey of the field achieved two things. First, he situated Darwin and his theory of natural selection scientifically and historically within “science” as a professional realm defined by theories like those in the model science physics. Second, he managed to write a book useful and accessible to both the non-specialist and the specialist. As a Penguin Pelican text, the imagined audience might have initially been aimed at the intellectually curious British public (and soon, international audiences).^11^ But when the book was in the last stages before publication, Maynard Smith realized he could make it appeal equally to professional biologists by including a reference list. Penguin agreed “anything that can make the book useful to the biologist proper as well as to the layman is all to the good.”^12^

This multi-layered audience initiated by Maynard Smith challenges the role of the popularizer in the Fleckian and diffusionist sense. Both perspectives argue that the migration of ideas between groups defined by level of expertise requires that popular knowledge is translated, simplified, even reified, expert knowledge. The process of making knowledge more accessible includes a removal from the uncertainties of scientific research into a world of “*[c]ertainty, simplicity, vividness*” (Fleck 1979, p. 115, italics in original; see also Brorson and Andersen 2001). Maynard Smith however explicitly did not omit details or difficulties while still addressing both “true laypeople” and biologists with varying levels and areas of expertise.

Even for evolutionary biologists, the book was of value. Evolutionary biology, as *The Theory of Evolution* aptly shows, is studied in various forms and fields: genetics, ethology, physiology, paleontology, embryology. No one could be an expert in each of these fields: “[e]ven the most specialized expert owes… many concepts, many comparisons, and even his general viewpoint” to popular science (Fleck 1979, p. 112). Maynard Smith’s text bridges both disciplines and levels of expertise. In doing so, he brought a large body of knowledge into circulation. The inclusion of scientific reference lists blurs the boundaries between popular science writing and science writing aimed at professionals, like handbooks and textbooks. Indeed, not only Maynard Smith himself used *The Theory of Evolution* for teaching; other scientists and students have found it helpful; textbook writers quoted it, and several universities used it as course textbooks.^13^

Maynard Smith and the publisher’s intention to make the book useful for both experts and non-experts was consequently realized in the actual audiences and their use of *The Theory of Evolution.* The text’s inclusion within the classroom meant the text reached those at the transition from non-expert to expert. This multiplicity of intended audiences mirrors the practices of nineteenth-century science writers, including Darwin himself. Evolutionary biology and other specific fields have used Darwin as an icon and role model. The aforementioned discontent of reviewers Fleming and Loewenberg of centennial era literature points to an even larger issue than simple failure to properly represent Darwin’s theory scientifically and historically. Evolutionary biology as a field was still emerging as a scientific discipline. As a burgeoning discipline, evolutionary biology needed Darwin’s work as their unifying theory to validate its professional status. Since Darwin’s important contribution, biologists worked towards getting recognition as scientists rather than amateurs, trying to rid “evolution” of an association with values and metaphysics by planting Darwin’s theory into the realm of the objective natural sciences. As the Centennial Celebration in America at the University of Chicago demonstrated, for evolutionary biologists “evolution by means of natural selection … had become a fact” (Smocovitis 1999, p. 279).

Betty Smocovitis has studied the Chicago celebrations in detail, noting “the supremacy of natural selection was a dominant theme … with panelists agreeing that genetical understanding of evolutionary mechanisms was leading to major advances” (1999, p. 298). The Darwin anniversary served both as a reassessment of recent developments and as a means to consolidate and reach out to a general audience. In this respect, some of the aims were not that different from those of *The Theory of Evolution*. At the same time, the celebrations were “part of an historical process of constructing disciplinary identities for evolutionary biologists and building a coherent identity for the collective community of scientists” (Smocovitis 1999, p. 321). These structures were only forming in the first half of the twentieth century. It took the efforts of many scientists to create umbrella-organizations, establish journals, and gather students so biology could become a unified and empirical science that has since been added to and expanded (Smocovitis 1992; Ruse 1996, 1999).

The history of the modern synthesis starts in the 1920s to 1930s. After the rediscovery of Mendel’s work in 1900, the geneticists (Mendelians) and selectionists (Darwinians) had seemed at odds with each other. In the 1930s, however, a group of biologists emerged who became known as the architects of the modern synthesis, combining the two approaches. The most prominent names are Maynard Smith’s mentor Haldane, R. A. Fisher, and the American Sewall Wright.^14^ They adopted “methodologies from the physical sciences to make evolution a more positive science” (Smocovitis 1992, p. 17). They were followed by other synthetic theorists working on and with these neo-Darwinian ideas. At the same time, biologists like Theodosius Dobzhansky, Ernst Mayr, George Gaylord Simpson, and Julian Huxley desired to professionalize evolutionary biology and to create a proper academic discipline in which they and others could work. These biologists were, as Ruse puts it, “under the spell of a metavalue.” They “wanted to move out of the museums and into the universities and to have all of the privileges and benefits of real researchers. They wanted their science to advance to the point where objectivity is a realizable aim” (Ruse 1999, p. 119).

Mathematics was one means to place evolutionary biology onto a more objective footing, introducing ways to measure and test natural selection. Haldane, Fisher, and Wright looked to the physical sciences for inspiration (Smocovitis 1992, pp. 20–22; see also Sheppard 1954, cited in Provine 1989, p. 478), and the Hardy–Weinberg equilibrium principle was one of those mechanisms which helped shape the body of evolutionary biology in a manner similar to that of physics. Thus, Maynard Smith’s support for mathematics not only relates to his training as an engineer,^15^ but also to his relationship with Haldane: “I’ve spent my life imitating Haldane” (Maynard Smith 1988, p. 128).

Maynard Smith was of the generation of evolutionary biologists who could build on the modern synthesis. At the same time, he advocated an increased use of mathematics in biology, following the lead of early population geneticists like Haldane while pushing against mathematical illiteracy in the wider biological community. The rejection of his early papers that integrated mathematics and biology highlights both his agenda and the scepticism towards the utility of mathematics in biology. Including mathematics in his popular writings does not seem to have been enough but it paved the way for an instantly successful textbook, *Mathematical Ideas in Biology.* Biology thus developed in the way Maynard Smith had advocated and his book officially “introduce[d] biologists from a broad spectrum of the subject to the use of mathematical modelling” (Charlesworth and Harvey 2005, p. 258).

The “little Penguin” was equally successful, with an inclusive audience of people outside of academia. It is in the tradition of evolutionary biologists like Dobzhansky, Mayr, Huxley, and Haldane, who all communicated their and their field’s ideas to audiences of non-specialists. It is also part of the effort to promote evolutionary biology as a science. As one reviewer said, “[b]oth the author and the publisher are to be praised for a book which helps to bring the scriptures of Wallace and Darwin from the realm of tropical visions supported by the dry bones of contention into the realm of science as both Harvey and Newton understood that term” (MacConaill 1959, p. 200). A decade later in 1969, Maynard Smith would indeed feel justified to say that “only in the study of evolution is there a body of biological theory in any way comparable to the theories of physics” (Maynard Smith 1972, p. 82; see also Smocovitis 1992, p. 55).

By bringing the theory of evolution by natural selection into the realm of science, Maynard Smith also managed to bring his readers into the realm of evolutionary biology. *The Theory of Evolution* “was my first introduction to John Maynard Smith and one of my first introductions to evolution,” wrote Dawkins (1993, p. xi), an appreciation shared by many others (Harper 2004; Partridge 2004; Charlesworth and Harvey 2005, p. 258).

## Conclusion

This microhistory of John Maynard Smith’s early popular writings, in particular his *Theory of Evolution*, highlights both continuities and discontinuities from nineteenth-century case studies that complicate the story of popular science in relation to science professionalization. On the one hand, his appeal to multiple audiences mirrors Darwin and Buckland. In fact, Maynard Smith actively placed himself in a line of science communicators who were both working scientists and writers of popular books. In the twentieth century, Richard Dawkins would follow in Darwin’s and Maynard Smith’s footsteps with his *Selfish Gene* (Topham 2009a, p. 18; Dawkins 1989, p. v). Lightman has in fact suggested that the practitioner-popularizer has come to dominate the field in the twentieth-century (2007, p. 419).

In contrast to the majority of successful practitioner-popularizers, however, Maynard Smith was an early-career researcher when he started popularizing science. It would be valuable to compare his trajectory to the professional status of the other twentieth-century writers Lightman mentions, such as E. O. Wilson, Richard Lewontin, Stephen J. Gould, Stephen Hawking, and Carl Sagan. It would also be interesting to know if there are differences between the sciences and their approaches to science popularization by practitioners. Many other lesser-known scientists who published one or two popular books in the early twentieth century were forgotten amid the mass of available material and because they were overshadowed by bigger names (Bowler 2006, p. 163). Maynard Smith, however, went on to become a successful and world-famous scientific researcher while keeping up his popularization work. Indeed, rather than harming his career, *The Theory of Evolution* was mentioned when Peter Medawar suggested Maynard Smith for the deanship at Sussex: “His Penguin on ‘The Theory of Evolution’ is absolutely first-rate, and I read it from cover to cover.”^16^

*The Theory of Evolution* reflects Maynard Smith’s multifaceted nature as a science communicator and the interconnectedness between his professional and popular work and publications. As we have seen, the text ties in with two types of professionalization: not only Maynard Smith’s own professional legacy, but also that of Darwin’s lasting impact. This latter feat happened through Maynard Smith’s contribution to the growing literature on Darwin and his theory of evolution by natural selection published during the centenary years of 1958 and 1959. Maynard Smith’s mission was to prove the theory of natural selection as true and central to our understanding of evolution and biology, presenting a distinct neo-Darwinian perspective. Another, more subtle contribution: he pushed his life-long conviction that mathematics plays a vital part in this understanding, which in Maynard Smith’s work—popular and professional—are clearly integrated. As the *Observer* noted upon the book’s republication by Canto editions:

‘Just a theory’, was President Ronald Reagan’s description of Darwinist evolution. Yes, but what a theory, one which has been robustly and repeatedly confirmed, using data and techniques that Darwin could not have imagined. This book – first published in 1958 but substantially revised with a long new introduction – is the best written introduction to the subject: well written, trenchant, an intellectual adventure story. (Anonymous 1993)

## Footnotes

1 “The Crafoord Prize 1999’,” 11 February 1999. https://www.crafoordprize.se/press_release/the-crafoord-prize-1999. Accessed 15 January 2019.

2 “Evolutionarily stable strategies.” The Great British Innovation Vote. https://webarchive.nationalarchives.gov.uk/20170405141907/http://www.topbritishinnovations.org/pastinnovations/evolutionarilystablestrategies. Accessed 15 January 2019.

3 Memorandum of Agreement, 16 February 1956. JMSA Add MS 86759, John Maynard Smith Archive, The British Library, London; hereafter cited as JMSA.

4 The Pelican Story. 2017. https://www.pelicanbooks.com/about. Accessed 23 July 2018. See also Anonymous 1938.

5 Abercrombie and Johnson, February 1953, DM 1952/614 A/02. Penguin Archives, Special Collections, University of Bristol; hereafter, PA. See also Abercrombie (1958, p. 9).

6 Abercrombie and Johnson, February 1953, DM 1952/614 A/02, PA.

7 Abercrombie to Glover, 19 September 1955, DM 1107/A433, PA.

8 THEORIES OF EVOLUTION by J. Maynard Smith (undated), DM 1107/A433, PA.

9 Maynard Smith to Yettram, 11 February 1958, DM 1107/A433, PA.

10 See White and Gribbin (2002), p. 223, which specifically mentions Stephen Hawking’s *Brief History of Time*, first published in 1988. This view appears to have shifted given the fact that a popular science book wholly dedicated to equations exists –*It Must Be Beautiful*, in which Maynard Smith has a chapter (Maynard Smith 2002).

11 It has been translated into at least five languages: French, Italian, Turkish, Spanish, and Portuguese.

12 Maynard Smith to Glover, 21 March 1958, DM 1107/A433, PA; Glover to Maynard Smith, 26 March 1958, DM 1107/A433, PA.

13 On teaching, see Maynard Smith to MacKeith, 4 September 1973, DM 1107/A433; on finding it helpful, see Sandon to Lutyens, 3 October 1959, DM 1952/614 A.02, PA. On textbook writers, see Stevick and Colver to Siddall, 10 May 1961, and Haagen-Smit to Siddall, 6 June 1963; Young to Ferguson, 18 July 1962; and Smith to Penguin Books, 26 July 1965, DM 1107/A433, PA. On course textbooks, see Ferraguti to Maynard Smith, 18 April 1983, JMSA Add MS 86575, PA; see also Toro and Santos 2004, p. 30.

14 Depew and Weber (1995) also add Sergei Chetverikov to that list.

15 Maynard Smith to Haldane, 6 October 1947, HP HALDANE/5/2/4/144. Haldane Papers, University College London.

16 Medawar to Fulton, 12 December 1963, Add MS 86575, JMSA.
